# Prognostic Implication of Preoperative Anemia in Redo Cardiac Surgery: A Single-Center Propensity-Matched Analysis

**DOI:** 10.3390/jcdd10040160

**Published:** 2023-04-06

**Authors:** Antonino Salvatore Rubino, Luca Salvatore De Santo, Antonio Pio Montella, Caterina Golini Petrarcone, Lucrezia Palmieri, Denise Galbiati, Nicola Galdieri, Marisa De Feo

**Affiliations:** 1 Cardio-Thoraco-Vascular Department, Division of Cardiac Surgery, Papardo Hospital, 98158 Messina, Italy; 2 Department of Translational Medical Sciences, University of Campania “Luigi Vanvitelli”, Monaldi Hospital, Via Leonardo Bianchi, 80131 Neaples, Italy; 3 Cardiovascular Department, Cardiac Surgery Unit of the IRCCS Humanitas Research Hospital, 20089 Rozzano, Italy; 4 Cardiac Intensive Care Unit, Monaldi Hospital, Via Leonardo Bianchi, 80131 Neaples, Italy

**Keywords:** anemia, reoperation, cardiac surgery, complications

## Abstract

Preoperative anemia has been associated with increased morbidity and mortality after cardiac surgery, but little is known about its prognostic value in the setting of redo procedure. A retrospective, observational cohort study of prospectively collected data was undertaken on 409 consecutive patients referred for redo cardiac procedures between January 2011 and December 2020. The EuroSCORE II calculated an average mortality risk of 25.7 ± 15.4%. Selection bias was assessed with the propensity-adjustment method. The prevalence of preoperative anemia was 41%. In unmatched analysis, significant differences between the anemic and nonanemic groups emerged in the risk for postoperative stroke (0.6% vs. 4.4%, *p* = 0.023), postoperative renal dysfunction (29.7% vs. 15.6%, *p* = 0.001), a need for prolonged ventilation (18.1% vs. 7.2%, *p* = 0.002), and high-dosage inotropes (53.1% vs. 32.9%, *p* < 0.001) along with both length of ICU and hospital stay (8.2 ± 15.9 vs. 4.3 ± 5.4 days, *p* = 0.003 and 18.8 ± 17.4 vs. 14.9 ± 11.1, *p* = 0.012). After propensity matching (145 pairs), preoperative anemia was still significantly associated with postoperative renal dysfunction, stroke, and the need for high-dosage inotrope cardiac morbidity. Preoperative anemia is significantly associated with acute kidney injury, stroke, and the need for high-dosage inotropes in patients referred for redo procedures.

## 1. Introduction

Preoperative anemia is increasingly encountered in the already complex comorbid profile of the relentlessly aging population referred for cardiac surgery. The inherent prognostic impact is still highly debated, since the level of evidence for its association with adverse outcomes is limited [1,2,3,4]. Indeed, most of the published studies suffer from a combination of single-center setting, retrospective design, small sample sizes, and focus on a single cardiac procedure and/or elective priority. Similarly, it is difficult to separate the risk of worsened outcomes because of preoperative anemia due to the risk associated with the augmented need for perioperative red blood cell (RBC) transfusion. Finally, the causal relation of different degrees of anemia severity is still under investigation, largely jeopardizing odds for the development and validation of preventive management algorithms. It remains difficult to determine whether anemia is a direct risk factor or a surrogate marker of the severity of associated comorbidities. As a matter of fact, current guidelines on patient blood management in cardiac surgery do not make an explicit recommendation on the management of preoperative anemia, and the preoperative risk stratification models that are most often used do not include preoperative anemia as a potential predictor of adverse outcome [5,6,7]. Despite this, a recent authoritative metanalysis of 114,277 patients found that anemia was associated with increased mortality, acute kidney injury, stroke, and infection [8]. Reoperations represent a significant proportion of modern cardiac surgery and are often associated with higher mortality and morbidity compared to first-time operations due to several inherent challenges (potential for reentry injuries, the combined/complex procedure, longer length of extracorporeal circulation support, worse comorbidity profile, and a higher need for RBC transfusions) [9,10]. Little is known of the prognostic value of anemia in this peculiar setting, since redo cardiac surgeries accounted for less than 5% of the cases in most of the articles on this topic. As part of our hospital’s ongoing continuous quality improvement program, this study aimed to investigate whether preoperative anemia is an independent determinant of adverse events in this augmented-risk patients’ subset. 

## 2. Materials and Methods

### 2.1. Study Sample, Design, and Aims

This single-center retrospective observational cohort study of prospectively collected data included all patients who underwent a redo cardiac surgical procedure in which the initial and subsequent procedures were performed through a median sternotomy (including ventricular-assist device implantations and transplantations) at the Department of Translational Medical Sciences, University of Campania “Luigi Vanvitelli,” located in an affiliated teaching hospital (V. Monaldi Hospital) between January 2011 and December 2020. Indications for redo procedures were based on a weekly heart team review process that involved surgeons, cardiologists, anesthesiologists, infectious disease specialists, and radiologists. The study population included 409 consecutive patients (mean age of 62 ± 12.7 years, octogenarians 5.6%, female sex 52.8%, diabetes 19.5%, chronic kidney disease 30%, urgent/emergent status 34.8%, third-time sternotomy 9.8%). The EuroSCORE II calculated an average mortality risk of 10.9 ± 11.7%. Study patients were segregated according to the presence or absence of preoperative anemia. The prevalence of preoperative anemia and its relationships with stroke, cardiac morbidity, and acute kidney injury (AKI) were obtained. Selection bias was assessed with the propensity-adjustment method. A secondary aim involving the prognostic implications of anemia severity was investigated. Research protocol was approved by the local ethics and research committee, which waived the need for informed consent.

### 2.2. Data Mining and Surgical and Clinical Care

At our institution, nearly 700 patients have cardiac surgery annually and are admitted to a dedicated 12-bed intensive care unit. Information from these patients is collected on a daily basis and maintained in the cardiac anesthesia database. Details on data mining, surgical procedure, and postoperative care have been reported extensively elsewhere [11,12,13]. Algorithms of patient blood management and patients’ compliance with current guidelines have been previously published [7,12,13]. All patients underwent multidetector CT (MDCT) scanning for planning surgical strategies [14]. This institutional protocol, as published in 2019, includes sternal compartment abnormalities, sternal/ascending aorta distance, assessment of the aorta (wall, diameters, lumen, valve), sternal/right ventricle distance, diaphragm insertion, pericardium and cardiac chambers, sternal/innominate vein distance, connection of existing coronary grafts to the predicted median sternotomy cut, graft patency and anatomic course, possible aortic cannulation and cross-clamping sites, and additional noncardiovascular significant findings [15]. As for institutional details of surgical technique, first, cardiopulmonary bypass was instituted prior to resternotomy in 151 cases (hemodynamic instability 108 and critical contiguity 43). Preferred peripheral cannulation sites were as follow: right common femoral artery in 137 cases and right axillary artery in 14 cases; the right common femoral vein was the preferred drainage route in 142 patients. Second, steel wires were removed only after division of the posterior table of the sternum by an oscillating saw. Electrocautery and/or scissors were employed to separate each side of the sternum from the underlying structures. Finally, the dissection plane was developed along the diaphragmatic surface and then up around the right atrium and towards the aorta, diligently avoiding penetration and extension beneath its adventitia. Left heart dissection along with isolation and control of patent internal thoracic artery grafts were usually achieved before CPB. Dissection of heart structures was kept to a minimum to perform the planned operation safely.

### 2.3. Baseline Data and Clinical Outcomes

All definitions were selected prospectively as part of the original study design. The preoperative hemoglobin level was prospectively defined as the lowest documented hemoglobin value among those measured at admission, during the preoperative period, or immediately before the induction of anesthesia. Gender-based definitions and the grading scale of anemia complied with statements by the World Health Organization (WHO) [16]. The change in kidney function was based on plasma creatinine concentration and was defined as the difference between baseline concentration and the highest concentration during the stay in the intensive care unit. Preoperative glomerular filtration rate (GFR) and smallest GFR during intensive care unit stay were calculated with the Modification of Diet in Renal Disease equation [17]. To classify preoperative kidney function, the 6-stages National Kidney Foundation Classification system was adopted [18]. For postoperative AKI, the RIFLE classification by the Acute Dialysis Quality Initiative Workgroup was referred to [19]. The main parameters considered in this study were creatinine and GFR, due to the heterogeneity of volemia status and diuretic use in this setting affecting the relationship between renal status and urine output. Cardiac morbidity was defined as the occurrence of a composite including myocardial infarction and/or heart failure. The diagnosis of myocardial infarction complied with the Third Universal Definition of Myocardial Infarction [20]. The diagnosis of heart failure required the use of either an intraaortic balloon pump or ECMO, the use of continuous high-dosage inotropic support for at least 24 h, or autopsy evidence of heart failure. Permanent neurological dysfunction (PND) was defined as a persistent loss of neurological function mainly caused by an ischemic event based on CT scan evidence of brain injury, which was classified, according to the extent, into focal (right or left hemispheric) or diffuse injury as described elsewhere [21].

### 2.4. Statistical Analysis

All statistical analyses were performed using SAS version 9.4 (SAS Institute, Cary, NC, USA). Continuous variables are reported as mean and SD, whereas categorical variables are reported as absolute number and percentage. Preoperative, operative and postoperative data were compared with unpaired *t*-test, chi-square, and the Kruskall-Wallis test. A nonparsimonious propensity score was calculated to select two groups of patients with similar baseline characteristics, with patients with preoperative anemia as the fixed group. The model included the following variables: age, gender, height, weight, diabetes, COPD, extracardiac vascular disease, LVEF, PAPs, serum creatinine, heart failure, surgical priority, aortic dissection, previous CABG, history of ischemic cardiomyopathy, history of preoperative stroke, preoperative mechanical ventilation, and diagnosis. One-to-one propensity score matching was performed using the greedy algorithm and a caliper of 0.2 of the standard deviation of the logit of the propensity score [22]. To evaluate the balance between the matched groups, paired sample *t*-test for continuous variables, the McNemar test for dichotomous variables, and analysis of the standardized differences after matching were used. Standardized differences lower than 0.10 were considered an acceptable imbalance between groups [23]. These tests were used to evaluate any difference in the adverse events of propensity score matched pairs. The association of preoperative anemia with the incidence of RIFLE F, the need for CVVH postoperative stroke, and cardiac morbidity was investigated with univariate logistic models, which were further adjusted for the obtained propensity score. In order to evaluate the prognostic significance of anemia severity, these analyses were repeated excluding patients presenting with severe and life-threatening hemoglobin concentrations. Statistical significance was set at an alpha level of 0.05.

## 3. Results

Among the whole operative cohort, 239 (58.4%) patients were classified as anemic. According to the WHO grading scale of anemia severity, patients were classified as follows: 160 (67.0%) presented with mild, 15 (6.3%) with moderate, 63 (26.3%) with severe, and only 1 (0.4%) with life-threatening anemia. Notably, among the worst two classes (64 patients), only 27 (42.2%) underwent surgery on an elective basis.

When segregating patients in the two groups of interest, several preoperative details differed between the groups (Table 1). Briefly, anemic patients were older with more comorbidities (diabetes, renal dysfunction, pulmonary hypertension, heart failure) and required more urgent operations. Similarly, distinctive underlying pathology referred patients for surgery. Such unbalance was adequately addressed in the propensity-matched population, with 145 pairs of patients with comparable preoperative risk profile.

When intraoperative details were considered, similar cannulation strategies and duration of surgery were observed in matched pairs. Conversely, anemic patients maintained intraprocedural a lower hemoglobin concentration, which translated into lower oxygen delivery throughout the procedure with an increased need for transfusion of packed red cells both during and after weaning from cardiopulmonary bypass (Table 2). 

This translated into a more pronounced postoperative impairment of renal function in patients with baseline lower hemoglobin concentrations. Accordingly, in both unmatched and matched analysis, anemic patients showed increased peak creatinine concentrations and lower filtration rates. Hence, the need for renal replacement therapy in the anemic group was doubled in unmatched analysis and was threefold higher in matched comparison. Similarly, stroke occurred more frequently in anemic patients, who also experienced a more prolonged duration of mechanical ventilation and cardiac morbidity (Table 3).

At univariate logistic analysis, preoperative anemia was independently associated with an almost fourfold increase in the odds of experiencing a significant worsening of renal function (AKI-Failure OR 3.69, 95%CI 1.90–7.18, *p* = 0.001; adjusted 2.11, 95%CI 1.02–4.39, *p* = 0.04) and with a sixfold increase in the odds of incurring into renal replacement therapy (OR 5.87, 95%CI 2.58–13.38, *p* < 0.001; adjusted 2.61, 95%CI 1.06–6.39, *p* = 0.036). Similarly, preoperative anemia was associated with incidences of de novo stroke (OR 8.10, 95%CI 1.03–63.90, *p* = 0.047; adjusted OR 6.01, 95%CI 1.01–51.84, *p* = 0.049), prolonged intubation (OR 2.88, 95%CI 1.46–5.67, *p* = 0.002; adjusted 1.88, 95%CI 1.27–3.96, *p* = 0.03), and cardiac comorbidities (OR 2.57, 95%CI 1.70–3.89, *p* < 0.001; adjusted OR 1.83, 95%CI 1.16–2.90, *p* = 0.01). Notably, some association of anemia with postoperative complications persisted even when the analysis was performed excluding patients presenting with WHO highest degrees of anemia (64 patients with at least severe anemia) (Table 4).

## 4. Discussion

This single-center retrospective observational cohort study of prospectively collected data including all patients who underwent a redo cardiac surgical procedure through a median sternotomy aimed to investigate whether preoperative anemia is associated with postoperative morbidity. Study patients were segregated according to the presence or absence of preoperative anemia. The prevalence of preoperative anemia and its relationship with acute kidney injury (AKI), stroke, cardiac morbidity, and prolonged ventilation were obtained. A significant association was found with all target events in the unmatched population. This association persisted after propensity matching. 

Nowadays, redo cardiac surgery accounts for approximately 15–20% of total cardiac surgical volume and is still associated with significantly higher morbidity and mortality than first-time operations. High-quality analysis also disclosed reoperation as an independent predictor of both short-term and long-term survival. Factors such as older age, female sex, preoperative renal failure, peripheral vascular disease, previous radiation to the chest or a sternal wound from multiple prior sternotomies, infection, endocarditis, priority, redo myocardial revascularization, and combined procedures are known independent predictors of worse outcomes. Little is known about the prognostic implications of preoperative anemia, a feature frequently encountered in the surgical population but up to now poorly explored in this setting. To the best of our knowledge, this is the first study to specifically address such an issue. In a real-world scenario (all comers, including all priorities and heart failure procedures, namely transplantation and ventricular assist device procedures), 58% of patients proved to be anemic, a figure in line with larger datasets [1,2,3]. Rates of target postoperative outcomes in this study favorably compare with contemporary series underlining the more demanding perioperative course of redo procedures [9,10]. The significant association of preoperative anemia with major postoperative complications as disclosed in the present study is largely confirmative of available evidence in unselected cardiac surgery cohorts and even with major noncardiac surgical series. These prognostic implications of anemia can be explained in several ways. First, since hemoglobin is a major determinant of tissue oxygen delivery, there is solid clinical and experimental evidence that oxygen supply to critical organs is compromised during the early stages of anemia and that this may trigger a dysoxia-related multiorgan dysfunction especially during cardiopulmonary bypass. Second, as clearly depicted in baseline patients’ features, anemics generally had poorer health, suffering from several comorbidities or frailty including malnutrition and sarcopenia. Altogether, these findings further support the addition of preoperative anemia to risk scoring models, as elegantly portended elsewhere [24]. Paradoxically, available risk stratification tools, such as the European System for Cardiac Operative Risk Evaluation, being based on multivariable logistic regression models, might have until now excluded anemia because of multicollinearity with several other comorbidities and risk factors carrying a higher prognostic impact. 

As for the risk of perioperative stroke, it has been reported in the range of 0.3% to 8% depending on the type of procedure. The pathogenesis is multifactorial. Nevertheless, there is strong evidence supporting a relationship between stroke and severity of hemodilution, which may cause both inadequate oxygen delivery and increased embolic load to the brain [25]. 

Cardiac surgery-associated acute kidney injury (CSA-AKI) occurs in nearly one-third of patients and represents one of the most important negative predictors of short- and long-term outcomes [26,27]. A recent authoritative article on patients referred for redo surgeries reported that AKI occurred in up to 54.3% of the cases, with renal replacement therapy required in 19.0% [28]. This compares favorably with data shown in our larger cohort. Pathophysiological mechanisms implicated in the genesis of CSA-AKI are multiple, but the synergistic role of anemia and transfusion is a key factor. There is convincing evidence that RBC transfusion is a risk factor for AKI, and on the other side, AKI acts as a major determinant of RBC need. Moreover, a growing body of evidence indicates that anemic patients are more susceptible to transfusion-related AKI than nonanemics [29,30,31]. In the peculiar setting of redo surgery, reentry injuries, extensive surgical dissection, and prolonged operative and cardiopulmonary bypass time significantly augment transfusion requirements. Indeed, in the authoritative analysis by Bianco et al., the need for blood product transfusion remained significantly elevated for the reoperative cohort, despite risk adjustment via propensity matching [9]. Intuitively, anemic patients in our study received more frequent red blood cell transfusions than nonanemic patients. The adjusted analysis in the present study disclosed that anemia is associated with a 2.5-fold increase in the risk for severe kidney injury and with a 3.3-fold increase in the risk for requiring renal replacement therapy. 

Additional information from our study is that the deleterious effects of preoperative anemia are not confined to the more severe cases. This finding is in good agreement with the conclusions of the authoritative study by Karkouti and colleagues demonstrating that even moderate anemia is associated with worse postoperative outcomes [32]. Intriguingly, these results somehow challenge the definition of preemptive treatment protocols [33]. A notion further underscored by the evidence is that the most severe degrees of anemia are related with higher surgical priorities. 

### Limitations

At least seven major study limitations should be considered for a thorough data interpretation. 

First, the single-center setting may be seen as a guarantee of a uniform process of care with special emphasis on transfusion triggers, but certainly it closely reflects the influence of specific standards of clinical and surgical practice and a unique patient population that may have led to one-sided results not readily transferable to other patient populations. Nevertheless, in this study the inclusion of all consecutive patients admitted for redo cardiac surgery (encompassing both high-priority procedures and massive blood transfusions) was intended to reproduce a real-world setting. This along with high-quality prospective data mining and the use of appropriate statistical processes for adjusting confounders has enhanced the chance of extrapolating these findings to other experiences. 

Second, the observational nature of our study prevents the definition of a causal relationship between anemia and the risk of postoperative adverse events. This applies also to the relation of the etiology and chronicity of this illness with outcomes. This is particularly important when considering the treatment of abnormal hemoglobin values. If these changes are reflective of underlying conditions, then severity of preoperative anemia may be a marker of risk and not a modifiable risk factor.

Third, the applicability of gender-based WHO criteria for anemia to the cardiac surgery setting has been recently questioned in favor of the calculated RBC mass. Indeed, the optimal preoperative hemoglobin levels to minimize perioperative transfusion and prevent postoperative complications remain largely undefined and might be more reflective of the type of surgery, along with sex, age, and individual perioperative hemoglobin level trajectories. 

Fourth, there is some evidence that the relationship between transfusion and adverse outcomes is affected by donor blood processing (leukodepletion) and storage duration [17]. Our study lacks any information about the length of RBC storage, though institutional donor blood processing entails leukodepletion. 

Fifth, the definition of AKI according to the “old” RIFLE criteria may deserve reconsideration. Definitions of AKI have evolved rapidly in recent years, from RIFLE (2004) through AKIN (2007) to KDIGO (2012). All three systems are complex and rely on non-SI units for creatinine. As reported in a recent authoritative review by Thomas and coworkers, “Systematic review has found that these definitions do not differ significantly in their performance” [34].

Sixth, the duration of follow-up was limited to the period of hospitalization. Thus, postdischarge complications could not be accounted for in our analysis.

Seven, larger cohorts are needed to further confirm our findings or highlight subtle intergroup differences. 

## 5. Conclusions

This single-center retroprospective observational study found that preoperative anemia is significantly associated with major postoperative morbidity after redo cardiac surgery. Further studies are warranted to determine whether preoperative low hemoglobin concentration is a marker of severity of illness or a modifiable risk factor. In this augmented-risk setting, development of peculiar patient blood management algorithms and especially a new preoperative risk scoring system encompassing preoperative anemia is strongly advised. 

## Figures and Tables

**Table 1:** Baseline details.

Characteristics	Overall Series				Propensity Score Matched Pairs			
	No Anemia (n = 170)	Anemia (n = 239)	*p*	Standardized Differences	No Anemia (n = 145)	Anemia (n = 145)	*p*	Standardized Differences
Age	60.5 ± 12.3	63.5 ± 12.1	0.015	0.24	61.8 ± 11.9	62.9 ± 12.7	0.40	0.09
Age ≥ 70	41 (24.1)	69 (28.9)	0.29	0.10	40 (28.0)	43 (30.0)	0.78	0.05
Female	79 (46.5)	114 (47.7)	0.81	0.03	72 (50.4)	71 (49.7)	>0.99	0.01
Height	165.6 ± 0.8	164.6 ± 9.2	0.30	0.11	164.8 ± 9.4	164.1 ± 8.8	0.51	0.08
Weight	74.8 ± 12.6	73.2 ± 14.3	0.25	0.13	73.7 ± 12.3	73.5 ± 14.6	0.93	0.01
BSA	1.85 ± 0.19	1.82 ± 0.20	0.17	0.15	1.83 ± 0.19	1.82 ± 0.19	0.73	0.04
BMI	27.2 ± 4.2	27.0 ± 5.0	0.56	0.06	27.0 ± 4.1	27.3 ± 5.4	0.58	0.06
Hemoglobin	14.3 ± 1.2	10.6 ± 1.6	<0.001	2.65	14.2 ± 1.1	10.9 ± 1.6	<0.001	2.29
Diabetes mellitus	23 (13.5)	57 (23.9)	0.009	0.26	23 (16.1)	24 (16.8)	>0.99	0.02
Serum creatinine	0.9 ± 0.3	1.1 ± 0.6	<0.001	0.46	0.9 ± 0.3	1.0 ± 0.4	0.36	0.07
eGFR	92.2 ± 57.3	73.7 ± 36.0	<0.001	0.39	86.6 ± 58.7	82.0 ± 37.0	0.39	0.10
0	33 (19.4)	97 (40.6)	<0.001	0.46	33 (22.8)	44 (30.3)	0.12	0.17
COPD	30 (17.7)	52 (21.8)	0.31	0.10	26 (18.2)	26 (18.2)	>0.99	0
Extracardiac arteriopathy	8 (4.7)	20 (8.4)	0.15	0.18	7 (4.9)	11 (7.7)	0.45	0.12
LVEF	54.1 ± 9.9	53.3 ± 0.1	0.39	0.08	53.5 ± 10.3	53.3 ± 9.8	0.88	0.02
PAPs	36.0 ± 13.3	39.3 ± 15.0	0.022	0.25	33.4 ± 13.3	38.1 ± 13.0	0.61	0.05
Pathology			<0.001	0.66			0.58	0.06
Endocarditis	4 (2.4)	48 (20.1)			4 (2.8)	5 (3.5)		
Failed repair/SVD	82 (48.2)	118 (49.4)			78 (54.6)	77 (53.9)		
End-stage HF	5 (2.9)	6 (2.5)			5 (3.5)	6 (4.2)		
Miscellaneous	79 (46.5)	67 (28.0)			56 (39.2)	55 (38.5)		
Aortic dissection	11 (6.5)	11 (4.6)	0.41	0.10	9 (6.3)	8 (5.6)	>0.99	0.03
Preoperative intubation	4 (2.4)	12 (5.0)	0.17	0.14	4 (2.8)	5 (3.5)	>0.99	0.04
Preoperative stroke	4 (2.4)	15 (6.3)	0.06	0.19	4 (2.8)	6 (4.2)	0.75	0.07
Ischemic cardiomyopathy	23 (13.5)	30 (12.6)	0.78	0.04	15 (10.5)	17 (11.9)	0.85	0.04
Previous CABG	27 (15.9)	31 (13.0)	0.41	0.10	21 (14.7)	22 (15.4)	>0.99	0.01
Heart failure	26 (15.3)	69 (28.9)	0.001	0.35	24 (16.8)	24 (16.8)	>0.99	0
Status			0.018	0.30			0.80	0.04
Election	134 (79.3)	162 (67.4)			112 (78.3)	112 (78.3)		
Urgent	22 (13.0)	57 (23.9)			20 (14.0)	19 (13.3)		
Emergency	13 (7.7)	21 (8.8)			11 (7.7)	12 (8.4)		

BSA: body surface area; BMI: body mass index; eGFR: estimated glomerular filtration rate; COPD: chronic obstructive pulmonary disease; LVEF: left ventricular ejection fraction; PAPs: systolic pulmonary artery pressure; SVD: structural valve deterioration; CABG: coronary artery bypass graft.

**Table 2:** Operative details and intraoperative outcomes.

Details	Overall Series			Propensity Score Matched Pairs		
	No Anemia (n = 170)	Anemia (n = 239)	*p*	No Anemia (n = 145)	Anemia (n = 145)	*p*
Presternotomy CPB	53 (31.2)	76 (31.9)	0.87	45 (31.5)	39 (27.5)	0.55
Preoperative arterial cannulation			0.33			0.47
No	110 (64.7)	147 (62.8)		93 (65.0)	96 (67.6)	
Femoral	56 (32.9)	81 (34.0)		47 (32.9)	40 (28.2)	
Axillary	3 (1.8)	10 (4.2)		3 (2.1)	6 (4.2)	
Subclavian	1 (0.6)	0		-	-	
Preoperative venous cannulation			0.50			0.82
No	114 (67.1)	152 (63.9)		96 (67.1)	98 (69.0)	
Femoral	56 (32.9)	86 (3)		47 (32.9)	44 (31.0)	
CPB [min]	153.9 ± 116.4	174.0 ± 86.1	0.045	160.0 ± 122.3	165.7 ± 84.9	0.51
XCT [min]	84.4 ± 44.4	99.3 ± 51.4	0.002	85.5 ± 44.2	94.5 ± 49.0	0.12
DHCA	11 (6.5)	11 (4.6)	0.42	9 (6.3)	9 (6.3)	>0.99
DHCA [min]	35.5 ± 29.9	42.5 ± 30.7	0.65	38.6 ± 33.0	47.1 ± 31.8	0.58
Cerebral perfusion			0.42			0.87
Kazui	7 (4.1)	10 (4.2)		6 (4.2)	8 (5.6)	
Axillary only	1 (0.6)	0		1 (0.7)	0	
Axillary + left carotid	1 (0.6)	0		-	-	
Cardioplegia			0.22			0.90
St. Thomas	149 (87.7)	205 (85.8)		125 (87.4)	125 (87.4)	
Custodiol	11 (6.5)	26 (10.9)		11 (7.7)	11 (7.7)	
Celsior	4 (2.4)	5 (2.1)		4 (2.8)	5 (3.5)	
Blood	2 (1.2)	0		-	-	
Length of surgery	314.3 ± 95.8	347.1 ± 120.7	0.003	318.8 ± 95.2	336.6 ± 123.6	0.19
Nadir DO2i	289.0 ± 76.9	240.2 ± 65.4	<0.001	284.4 ± 78.0	246.7 ± 62.8	<0.001
Nadir DO2i ≤ 262	47 (28.0)	137 (57.8)	<0.001	42 (29.6)	74 (52.5)	<0.001
Nadir Hb	8.7 ± 1.4	7.2 ± 1.1	<0.001	8.6 ± 1.4	7.4 ± 1.1	<0.001
Nadir Hct	26.4 ± 4.1	21.8 ± 3.3	<0.001	26.0 ± 4.1	22.5 ± 3.3	<0.001
PRC during CPB	0.4 ± 1.1	1.9 ± 2.0	<0.001	0.5 ± 1.2	1.5 ± 2.0	<0.001
PRC after CPB	0.4 ± 0.9	1.0 ± 1.5	<0.001	0.4 ± 1.0	0.8 ± 1.3	<0.001
PRC total	1.2 ± 2.0	3.4 ± 3.3	<0.001	1.3 ± 2.0	2.8 ± 3.1	<0.001

CPB: cardiopulmonary bypass; XCT: cross-clamping time; DHCA: deep hypothermic circulatory arrest; DO2i: indexed oxygen delivery; Hb: haemoglobin; Hct: haematocrit; PRC: packed red cells.

**Table 3:** In-hospital results.

Details	Overall Series			Propensity Score Matched Pairs		
	No Anemia (n = 170)	Anemia (n = 239)	*p*	No Anemia (n = 145)	Anemia (n = 145)	*p*
Peak creatinine	1.3 ± 0.7	1.7 ± 1.0	<0.001	1.3 ± 0.7	1.5 ± 0.8	0.049
Nadir eGFR	73.0 ± 44.4	57.1 ± 38.4	<0.001	68.1 ± 40.2	60.1 ± 33.2	0.08
AKI			0.001			0.06
No	108 (64.7)	123 (54.4)		89 (62.7)	81 (59.6)	
Risk	33 (20.0)	36 (15.9)		31 (21.8)	22 (16.2)	
Injury	14 (8.4)	17 (7.5)		12 (8.5)	10 (7.4)	
Failure	12 (7.2)	50 (22.1)		10 (7.0)	23 (16.9)	
CVVH	7 (4.2)	46 (20.4)	<0.001	6 (4.2)	19 (14.0)	0.006
AKI injury-failure	26 (15.6)	67 (29.7)	0.001	22 (15.5)	33 (24.3)	0.10
Cardiac morbidity	59 (34.7)	133 (55.7)	<0.001	50 (34.5)	67 (46.2)	0.056
ECMO	3 (1.8)	11 (4.6)	0.12	2 (1.4)	3 (2.1)	>0.99
IABP	8 (4.7)	18 (7.5)	0.25	7 (4.8)	9 (6.3)	0.80
High-dose inotropes	56 (32.9)	127 (53.1)	<0.001	46 (32.2)	67 (46.9)	0.011
Simdax	8 (4.7)	29 (12.3)	0.02	8 (5.6)	18 (12.6)	0.013
Prolonged mechanical ventilation	12 (7.2)	41 (18.1)	0.002	9 (6.3)	20 (14.7)	0.035
Tracheostomy	3 (1.8)	10 (4.4)	0.15	2 (1.4)	7 (5.2)	0.18
Readmission to ICU	4 (2.4)	10 (4.4)	0.38	4 (2.8)	5 (3.4)	0.82
Bleeding requiring reoperation	6 (3.6)	6 (2.7)	0.59	4 (2.8)	4 (2.8)	>0.99
Postoperative stroke	1 (0.6)	10 (4.4)	0.023	1 (0.7)	7 (5.1)	0.0097
ICU stay	4.3 ± 5.4	8.2 ± 15.9	0.003	4.6 ± 5.8	7.3 ± 16.5	0.08
Postoperative stay	14.9 ± 11.1	18.8 ± 17.4	0.012	15.6 ± 11.7	17.9 ± 16.5	0.21

eGFR: estimated glomerular filtration rate; ICU: intensive care unit; AKI: acute kidney injury; CVVH: continuous veno-venous hemofiltration; ECMO: extra corporeal membrane oxygenation; IABP: intraaortic balloon pump.

**Table 4:** Association of mild and moderate anemia with adverse postoperative outcomes (excluding patients with highest degree of anemia).

Outcomes	Unadjusted			Propensity Score Adjusted		
	OR	95%CI	*p*	OR	95%CI	*p*
AKI-Failure	3.23	1.61–6.48	0.001	2.07	1.02–4.37	0.047
CVVH	5.10	2.18–1.94	<0.001	2.62	1.05–6.54	0.039
Stroke	7.13	1.86–58.58	0.032	6.97	1.79–60.91	0.047
Prolonged mechanical ventilation	2.77	1.36–5.62	0.005	1.95	1.91–4.16	0.046
Cardiac mordibity	2.18	1.40–3.38	<0.001	1.78	1.11–2.86	0.017

AKI: acute kidney injury; CVVH: continuous veno-venous hemofiltration.

## Data Availability

The data presented in this study are available on request from the corresponding author.
